# Perillaldehyde inhibits oxidative stress, NLRP3-mediated inflammation and fibrosis in diabetic nephropathy through regulating HMOX1

**DOI:** 10.1186/s41065-026-00657-y

**Published:** 2026-03-06

**Authors:** Wen Hao, Chunhua Liu, Jing Feng, Zhanliang Ren

**Affiliations:** 1 Department of Endocrinology, the Affiliated Hospital of Northwest University, Xi’an No.3 Hospital, Xi’an, Shaanxi Province 710000 China; 2 Department of General Surgery, the Affiliated Hospital of Northwest University, Xi’an No.3 Hospital, Xi’an, Shaanxi Province 710000 China; 3 Department of Cardiothoracic Surgery, the Affiliated Hospital of Shaanxi, University of Traditional Chinese Medicine, Xianyang, Shaanxi Province 712000 China

**Keywords:** Diabetic nephropathy, perillaldehyde, HMOX1, oxidative stress, inflammation, fibrosis

## Abstract

Diabetic nephropathy (DN) is a frequently occurred complication of diabetes mellitus (DM) worldwide. Perillaldehyde is a natural terpenoid compound exerted beneficial effects on diabetic cardiomyopathy (DCM), which is another important part of DM complication. However, the effect of perillaldehyde on DN has not been reported. In the present study, we demonstrated that perillaldehyde prevented ROS-driven oxidative stress, with decreased ROS and MDA levels and increased GSH level in high glucose (HG)-treated HK-2 cells. Perillaldehyde also reversed HG-induced NLRP3-mediated inflammation and fibrosis in HK-2 cells, as evidenced by decreased pro-inflammatory cytokines and fibrogenic factors. Furthermore, we observed that perillaldehyde directly targeted HMOX1 and regulated its expression in HK-2 cells. Knockdown of HMOX1 prevented the effects of perillaldehyde on HG-treated HK-2 cells, implying that perillaldehyde exerted its effects via regulating HMOX1. In a high fat diet (HFD)/streptozocin (STZ) mouse model, perillaldehyde also exerted renoprotective, antioxidant, anti-inflammatory and anti-fibrotic activities. In conclusion, our study clearly demonstrated the beneficial effects of perillaldehyde on DN both in vivo and in vitro, which indicated that perillaldehyde might serve as a potential application in treating or preventing DN.

**Supplementary Information:**

The online version contains supplementary material available at 10.1186/s41065-026-00657-y.

## Introduction

Diabetic nephropathy (DN) is a frequently occurred complication of diabetes mellitus (DM) worldwide and remains the leading cause of end-stage renal disease (ESRD) [1]. Over the past decade, the incidence and prevalence of DN are increasing, especially in developing countries [1]. The pathogenesis of DN is not exactly clear, therefore the therapeutic outcomes is still poor. Until now, there is no obvious improvement in clinical strategy for preventing the progression of DN. Exploring the pathogenic mechanisms of DN is very important to improve and/or develop new strategies for preventing and treating DN [2].

Recently, improved understanding of DN pathogenesis has shown that many pathways and mediators, including oxidative stress and inflammatory processes, are implicated in the onset of DN development [3]. Mechanistically, chronic hyperglycemia induces excess production of reactive oxygen species (ROS), which remains the primary initiator of renal and systemic damage in DM and its complications [4]. The overproduction of ROS reduces antioxidant capacity and induces oxidative stress in different types of renal cells, such as endothelial cells, mesangial cells, tubular epithelial cells and podocytes [3]. The oxidative damage stimulates the release of inflammatory mediators and cytokines and activates inflammatory response, thereby exacerbating oxidative damage [5]. Then the formation of oxidative stress-inflammation cycle may trigger various pathological processes, such as epithelial-mesenchymal transition, extracellular matrix accumulation, renal fibrosis, glomerulosclerosis, and proteinuria [6]. It has been proven that targeting oxidative stress or inflammation may delay the progression of DN. Increasing antioxidant and anti-inflammatory agents are considered to be promising candidates for DN therapy [6–9]. NOD-like receptor family pyrin domain-containing 3 (NLRP3) inflammasome plays a pivotal role in the pathophysiology of diabetes and its complications including DN, which contains sensor protein NLRP3, the adaptor protein apoptosis-associated speck-like protein containing a CARD (ASC) and the effector protein caspase-1 [10, 11].

**Fig. 1:**
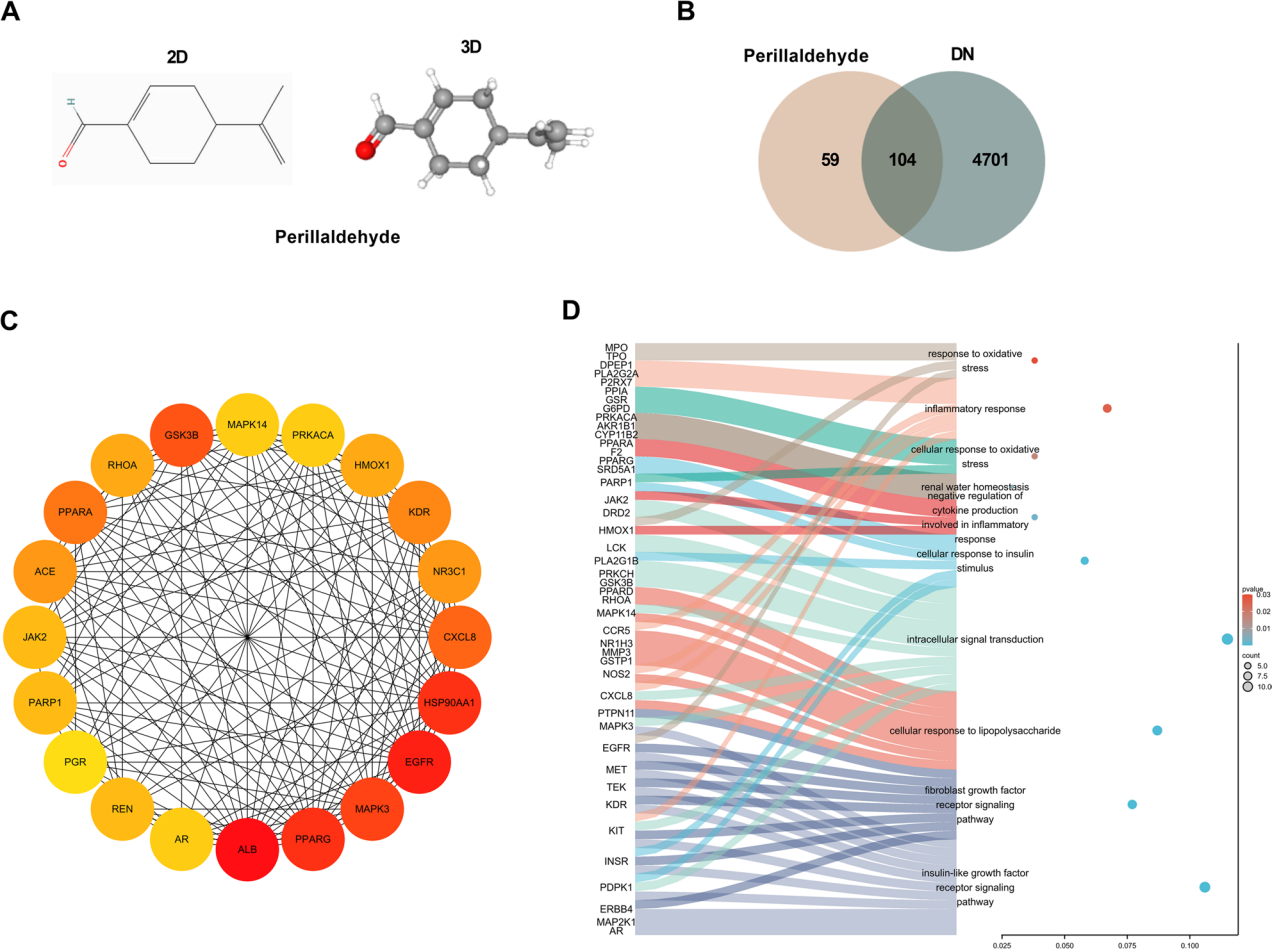
The network pharmacology of perillaldehyde in DN. **A** Chemical structure of perillaldehyde. **B** Venn diagram indicating the 104 intersection genes between targets of Perillaldehyde (163) and targets of DN (4805). **C** PPI networks constructed by String and Cytoscape. **D** GO BP analysis by DAVID

Perillaldehyde (its chemical structure was shown in Fig. 1A) is a natural terpenoid compound extracted from *Perilla frutescens*, which is commonly used as a leafy vegetable, food additives and traditional medicine concoctions [12]. The small lipophilic compounds can be taken up by passive diffusion. In recent years, perillaldehyde has gained a lot of attention due to its multitude of human health benefits including neuroprotective [13, 14], antioxidant [15], anti-inflammatory [16], antitumor [17], antiatherosclerotic [18], antifungal and other microbial activities [19–21]. Biological analyses of perillaldehyde also reveal that perillaldehyde exerts beneficial effects on diabetic cardiomyopathy (DCM), which is an important part of diabetes mellitus (DM) complications [22, 23]. In addition, perillaldehyde also exhibits protective effect on sepsis-associated acute kidney injury [24]. However, the effect of perillaldehyde on DN has not been reported.

HMOX1 is a critical gene involved in cellular stress response. It is evident that protein HMOX1, also known as HO-1, catalyzes the multistep oxidation of heme [39]. HMOX1 is a downstream gene regulated by Nrf2, which has been clearly established to be closely linked to oxidative stress [25]. Moreover, HMOX1 has been suggested to be associated to inflammation in DN [26, 27]. Based on an in-silico analysis, HMOX1 might act as a target of perillaldehyde, which has not been reported.

In the present study, we demonstrated that perillaldehyde presented protective effect on DN both in vivo and in vitro. Furthermore, we observed that its antioxidant, anti-inflammatory and anti-fibrotic activities contributed to its positive role in DN.

## Methods and materials

### Data preprocessing based on the online datasets

The targets of perillaldehyde were downloaded from HERB (10 genes) (http://herb.ac.cn/), SwissTargetPrediction (100 genes) (http://www.swisstargetprediction.ch/), and PharmMapper (63 genes) (http://www.lilab-ecust.cn/pharmmapper/). This list was filtered to remove duplicates to obtain 163 genes. The targets of DN were obtained from GeneCards (4805 genes) (https://www.genecards.org/). The online service String (https://string-db.org/) was utilized to construct the protein-protein interaction (PPI) networks, and visualized using Cytoscape 3.10.3. The network topology was analyzed by using Cytoscape’s built-in tools and identified hub targets with cytoHubba. The core targets in the PPI network were screened according to the top 20 ranked genes analyzed by Degree algorithm. The DAVID database (https://davidbioinformatics.nih.gov/) was used for gene ontology (GO) biological process (BP) analysis.

### Cell culture and treatments

The human renal tubular epithelial (HK-2) cells (ATCC, Manassas, VA, USA) were cultured in DMEM (Gibco Laboratories, Grand Island, NY, USA) supplemented with 10% FBS (Gibco Laboratories) and 1% penicillin-streptomycin (Gibco Laboratories). The cells were maintained at 37 °C in a humidified atmosphere with 5% CO_2_. After incubation in serum-free medium overnight, the HK-2 cells were initially pretreated with 0, 20, 40, or 80 µM perillaldehyde (dissolved in DMSO; MedChemExpress, Monmouth Junction, NJ, USA) for 4 h followed by incubation with a final concentration of 30 mM D-glucose for 48 h referring to previous reports [28, 29]. The concentration of perillaldehyde was selected according to a previous study [23]. Cells in the control group were incubated in normal glucose (5.5 mM D-glucose). For positive control group, cells were pretreated with antioxidant (ROS scavenger) N-acetylcysteine (NAC; 10 mM; Beyotime, Shanghai, China).

SiRNA negative control (si-con) and siRNA against HMOX1 (si‐HMOX1) were synthesized by Genepharma (Shanghai, China). When reaching 50–70% confluence in 6-well plates, HK-2 cells were transfected with si‐con or si‐HMOX1 (20 nM) using 10 µL Lipofectamine™ 2000 liposome (Invitrogen, Carlsbad, CA, USA) for 6 h according to the instructions. Cells were allowed to recover for 18 h and then collected for the indicated treatment. The siRNA had no negative influence on the viability of HK-2 cells.

### Cell viability assay

Cell viability was evaluated using the cell counting kit-8 solution (CCK-8) assay (Dojindo, Kumamoto, Japan). Briefly, HK-2 cells were seeded at a density of 1 × 10^4^ cells per well in 96-well plates. After various treatments, 10 µl CCK-8 solution was added to the cells and incubated for 2 h. Cell viability was measured by measuring the OD at 450 nm using a microplate reader (Molecular Devices, San Jose, CA, USA).

### Detection of oxidative stress indicators

HK-2 cells were added in 6-well plates (2 × 10^5^/well) and subjected to the indicated treatment. For reactive oxygen species (ROS) assay, cells were incubated with cellular ROS detection assay kit (Abcam, Cambridge, MA, USA) according to the manufacturer’s instructions, and nucleus were stained with DPAI (Beyotime). ROS level was detected with a fluorescence microscope (Olympus, Tokyo, Japan), and shown as fold change of the control group.

The oxidative stress-related factors including malondialdehyde (MDA) level, and GSH level were evaluated using commercial kits (Nanjing Jiancheng Bioengineering Institute, Nanjing, China) following the manufacturer’s instructions.

### Western blot

HK-2 cells were added in 6-well plates (2 × 10^5^/well) followed by the indicated treatment. After preparation of total proteins by homogenizing from HK-2 cells and kidney tissues (15 mg:150 µl) using radioimmunoprecipitation lysis buffer (Beyotime), protein concentration was measured by using BCA protein assay kit (Beyotime). western blot analysis was performed using twenty µg total protein to detect the expression levels of NLRP3, ASC, p-p65, p65, cleaved caspase-1, GSDMD-N, E-cadherin, α-SMA, TGF-β1, collagen IV, fibronectin, HMOX1 as described previously [30]. Primary rabbit antibodies (dilution: 1:1000) directed against these proteins and appropriate goat anti-rabbit secondary antibodies (dilution: 1:10000) were obtained from Cell Signaling Technology (Danver, MA, USA) or Abcam. Finally, the protein bands were developed using an enhanced chemiluminescence (ECL) kit (Beyotime) and visualized by Image-Quant LAS 400 camera system.

### ELISA

HK-2 cells were placed in 6-well plates (2 × 10^5^/well). After indicated treatments, supernatants of HK-2 cells cultured medium were collected. The kidney tissues (10 mg:100 µl) was homogenized and supernatants were collected after centrifugation at 12,000 rpm for 10 min. Levels of pro-inflammatory cytokines including TNF-α, IL-6, and IL-1β were measured using commercial ELISA kits (R&D, Minneapolis, MN, USA), according to the manufacturer’s instructions. The optical density (OD) of samples was measured using a Microplate Reader (Molecular Devices) at 450 nm.

### Molecular docking and molecular dynamics simulation

Structural models for perillaldehyde (Compound CID: 16441) and HMOX1 (ID: P09061) were respectively obtained from PubChem database (https://pubchem.ncbi.nlm.nih.gov) and Protein Data Bank (PDB) (www.rcsb.org/pdb). Molecular docking study was performed to assess the interaction between perillaldehyde with HMOX1 structure as previously described [31]. The docking results were visualized and analyzed by using PyMOL 3.1 (Schrödinger, San Diego, CA, USA; http://www.pymol.org/) and AutoDock Vina program 1.1.2. The molecular dynamics simulation was performed by using GROMACS 2022.2 as reported previously [32].

### Pull down assay

The binding between perillaldehyde and HMOX1 was analyzed using a pull-down assay kit (Thermo Fisher Scientific, Waltham, MA, USA). Biotinylated perillaldehyde was incubated with streptavidin–agarose beads at 4 °C for 30 min, and biotin alone was used as negative control. Cell lysates were incubated with streptavidin–agarose beads for 24 h, then samples were collected and used for western blot assay.

### Animal experiments

A total of 24 C57BL/6 mice (male, aged eight-week-old) were purchased from Model Animal Research Center of Nanjing University (Nanjing, China). The type 2 diabetes mellitus (T2DM) model was induced by high-fat diet and streptozotocin (HFD/STZ). These mice were divided randomly into two groups: normal control group (*n* = 8), mice were fed with a normal diet (10% kcal fat) sustainably for 10 weeks; HFD/STZ group (*n* = 16), mice were fed with a high fat diet (60% kcal fat, 20% kcal protein, 20% kcal carbohydrates) for 4 weeks, followed by intraperitoneal injection with STZ (100 mg/kg body weight). Two weeks after STZ injection, mice with fasting plasma glucose levels (> 11.1 mM) were considered as diabetic cases and selected for further experiments. Actually, 16 diabetic mice have been selected and randomly allocated into two groups: HFD/STZ group and HFD/STZ+perillaldehyde group. Mice in HFD/STZ+perillaldehyde group were given perillaldehyde (100 mg/kg per day) for 4 weeks. The dosage of perillaldehyde was selected as reported previously [17, 24]. No death was occurred during the study. The animal experiments were approved by the Ethics Review Committee of the Affiliated Hospital of Northwest University (Xi’an, China) (ethical approval number: No. NWU-AWC-2024061702 M).

At the end of the experiments, blood samples (~ 500 µl) were collected via rapid cardiac puncture. The fast blood glucose, blood urea nitrogen (BUN), and insulin levels were then determined to evaluate kidney function. The homeostasis model assessment of insulin resistance (HOMA-IR) and β-cell function (HOMA-β) were analyzed according to a previous report [33]. All mice were then anesthetized with 2% isoflurane and euthanized by cervical dislocation. Kidneys were collected and weighed, then the kidney weight (KW)/body weight (BW) index was calculated. A portion of kidneys was fixed with 4% paraformaldehyde and cut into sections at a thickness of 5 μm. Hematoxylin and eosin (H&E), periodic acid-Schiff (PAS) and Masson’s trichrome staining were performed to evaluate collagen deposition kidney injury and fibrosis as previously described [34]. The remaining part of kidneys was quickly frozen in liquid nitrogen, and then stored at − 80 °C for subsequent experiments.

### Statistical analysis

All statistical analysis in this study was performed using the SPSS 22.0 software (SPSS Inc., Chicago, IL, USA). The one-way analysis of variance (ANOVA) with Tukey’s post hoc analysis was adopted for multiple comparisons. *p* < 0.05 was defined as statistically significant.

## Results

### The network pharmacology of perillaldehyde in DN

Venn diagram (Fig. 1B) presented the 104 intersection genes between targets of perillaldehyde (163) and targets of DN (4805). Then the String and Cytoscape were used to construct the PPI networks with the top 20 ranked genes analyzed by Degree algorithm, and HOMX1 ranked 13th in the Degree network (Fig. 1C). As shown in Fig. 1D, these proteins are closely associated with either oxidative stress or inflammation. Importantly, HMOX1, as a critical antioxidant factor, has been found to be closely related to both oxidative stress and inflammation. Thus, HMOX1 was selected as a potential target of perillaldehyde in this study.

### Perillaldehyde inhibits oxidative stress in HG-treated HK-2 cells

**Fig. 2:**
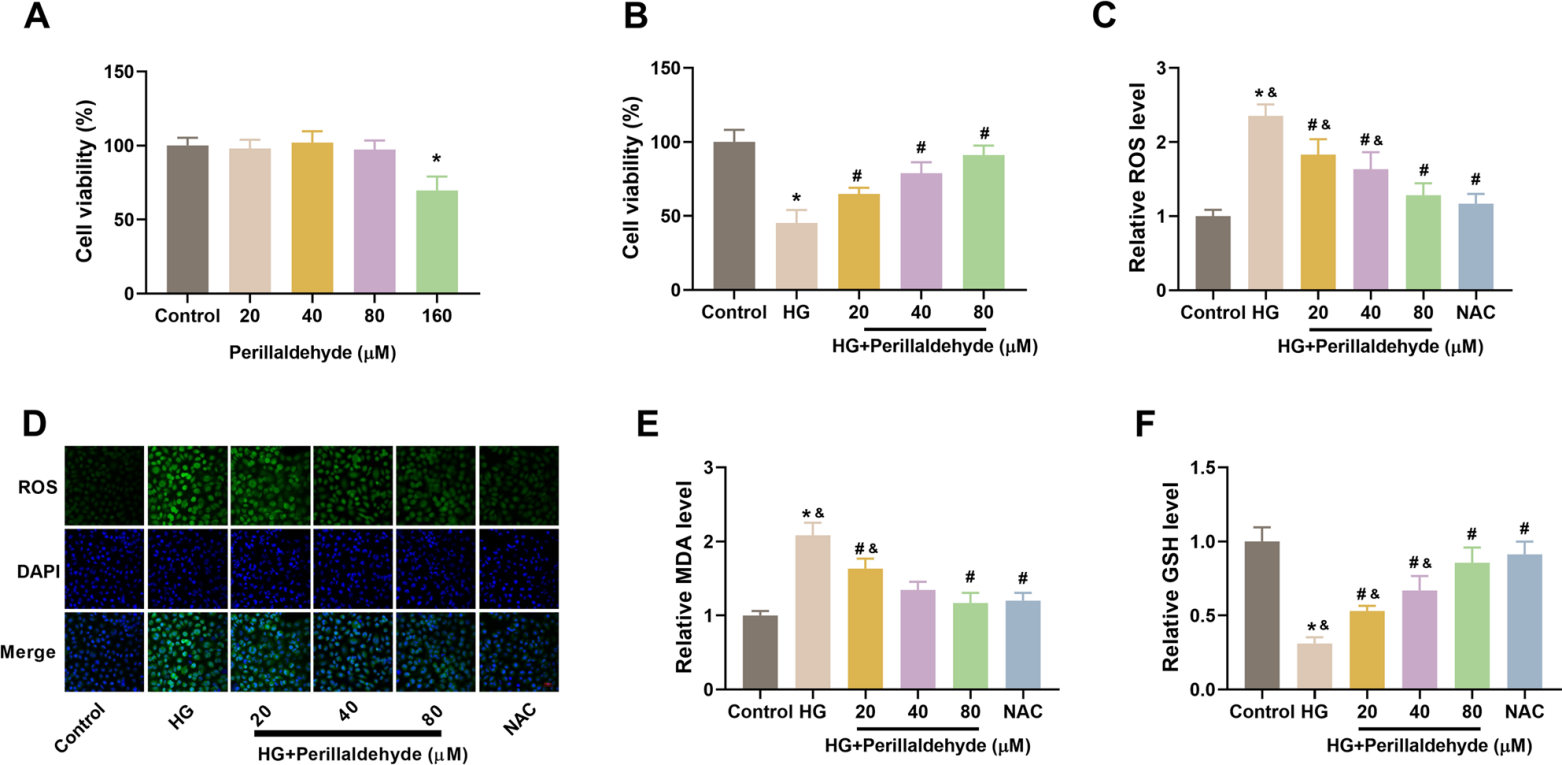
Perillaldehyde inhibits HG-induced oxidative stress in HK-2 cells. **A** CCK-8 assay was used to select perillaldehyde concentration. **B** CCK-8 assay was performed after pretreatment with perillaldehyde (20, 40, or 80 µM) for 2 h and the following 30 mM D-glucose stimulation. HK-2 cells were pretreated with perillaldehyde (0, 20, 40, or 80 µM) or NAC (10 mM), followed by incubation with 30 mM D-glucose for 48 h. The oxidative stress-related indicators including ROS (**C**-**D**), MDA (**E**) and GSH (**F**) were determined. NAC was used as a positive control. Scale bar = 20 μm. *N* = 3. *p < 0.05 vs. control group, ^#^*p* < 0.05 vs. HG group, ^&^*p* < 0.05 vs. NAC group

As shown in Fig. 2A, perillaldehyde did not affect cell viability of HK-2 cells, except for the concentration of 160 µM. The concentrations of 20, 40, and 80 µM without cytotoxicity were selected for the following studies. In addition, we observed that perillaldehyde (20, 40, or 80 µM) prevented the reduction in viability induced by HG in HK-2 cells (Fig. 2B). Furthermore, to investigate the effect of perillaldehyde on oxidative stress in HK-2 cells, we assessed the effects of perillaldehyde on ROS level, MDA content and GSH level in HK-2 cells under HG stimulation. ROS level in HG induction group was significantly higher than that of control group. Treatment with perillaldehyde (20, 40, or 80 µM) or NAC attenuated the increased ROS level in HG-treated HK-2 cells (Figs. 2C-D). In addition, HG also caused obvious increase in MDA content and decrease in GSH level, which were reversed by perillaldehyde (20, 40, or 80 µM) or NAC (Figs. 2E-F).

### Perillaldehyde inhibits NLRP3-mediated inflammation in HG-treated HK-2 cells

**Fig. 3:**
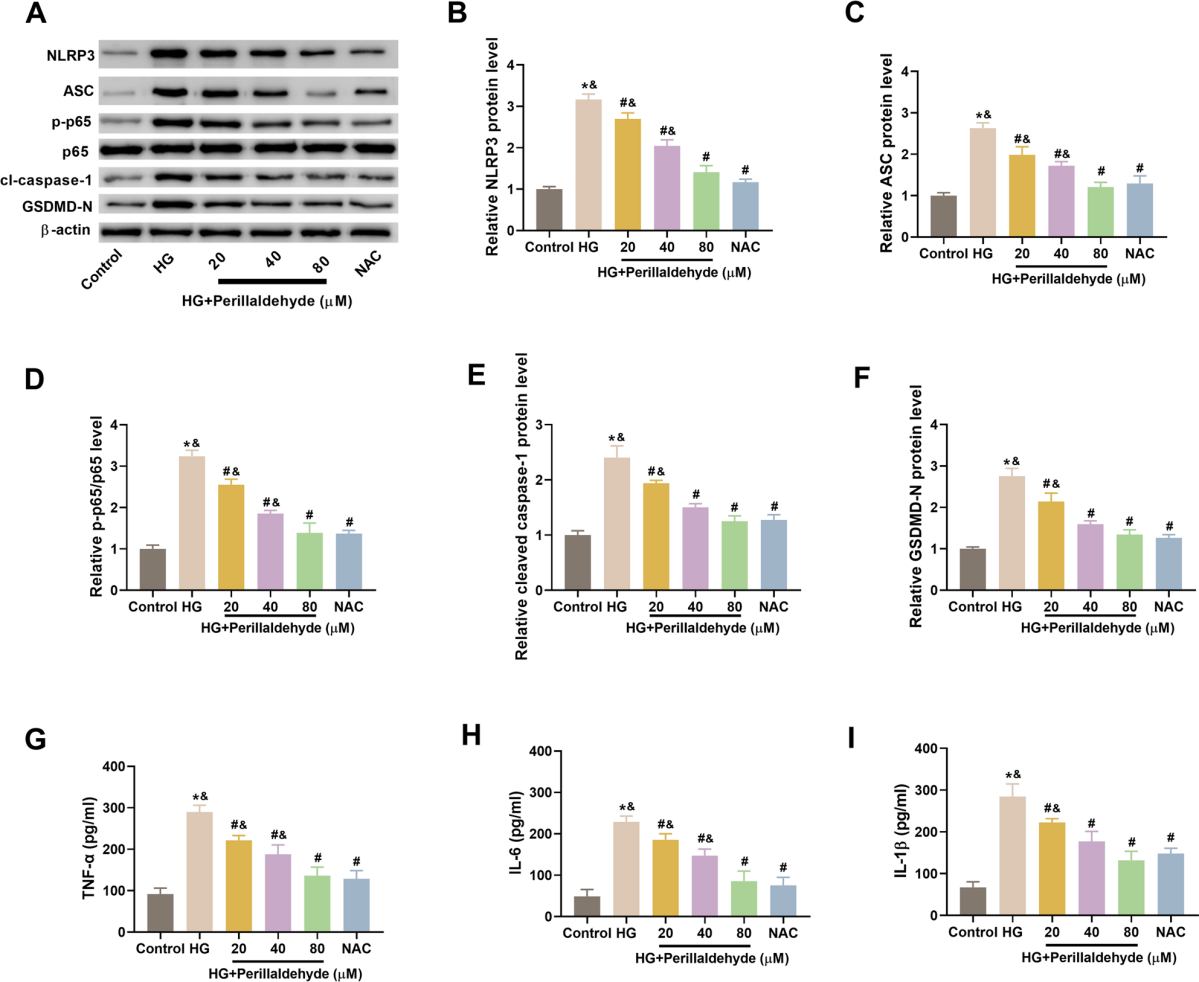
Perillaldehyde inhibits NLRP3-mediated inflammation in HG-treated HK-2 cells. **A**-**F** Western bot analysis for detecting the expression levels of NLRP3, ASC, cleaved caspase-1. GSDMD-N, and p-p65/p65. **G**-**I** ELISA for assaying the secretion levels of TNF-α, IL-6, and IL-1β in cell culture of HK-2 cells. NAC was used as a positive control. *N* = 3. *p < 0.05 vs. control group, ^#^*p* < 0.05 vs. HG group, ^&^*p* < 0.05 vs. NAC group

Next, we evaluated the effect of perillaldehyde on NLRP3-mediated inflammation through detecting the related protein expression levels in NLRP3 inflammasome complex. As presented in Figs. 3A-F, HG induction caused significant increase in the expression levels of NLRP3, ASC, cleaved caspase-1, GSDMD-N and p-p65, while pretreatment with perillaldehyde or NAC prevented the increase of these proteins. Moreover, the levels of TNF-α, IL-6, and IL-1β were markedly increased in HG-treated HK-2 cells, which were significantly downregulated by perillaldehyde or NAC (Figs. 3G-I).

### Perillaldehyde inhibits fibrosis in HG-treated HK-2 cells

**Fig. 4:**
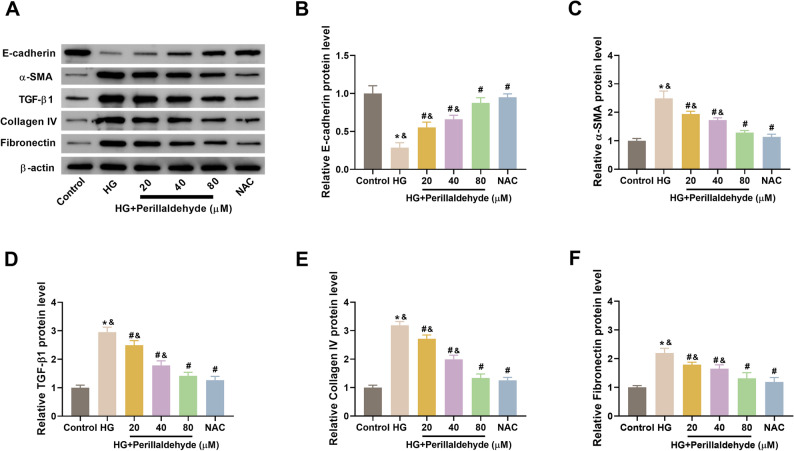
Perillaldehyde inhibits HG-induced fibrosis in HK-2 cells. Western blot analysis was conducted for detecting the expression levels of fibrosis-related proteins including E-cadherin, α-SMA, TGF-β1, collagen IV, and fibronectin. **A **Representative images of western blot. **B**-**F** Relative expression levels of E-cadherin, α-SMA, TGF-β1, collagen IV, and fibronectin. NAC was used as a positive control. *N* = 3. *p < 0.05 vs. control group, ^#^*p* < 0.05 vs. HG group, ^&^*p* < 0.05 vs. NAC group

To observe the effect of perillaldehyde on fibrosis, the fibrosis-related proteins including E-cadherin, α-SMA, TGF-β1, collagen IV, and fibronectin were measured. Figures 4A-F, HG induction caused significant decrease in E-cadherin expression, as well as increase in α-SMA, TGF-β1, collagen IV, and fibronectin expression. Pretreatment with perillaldehyde or NAC attenuated the effects of perillaldehyde on these fibrosis-related proteins, implying that perillaldehyde reversed HG-induced fibrosis in HK-2 cells.

### HMOX1 mediates the effect of perillaldehyde on oxidative stress

**Fig. 5:**
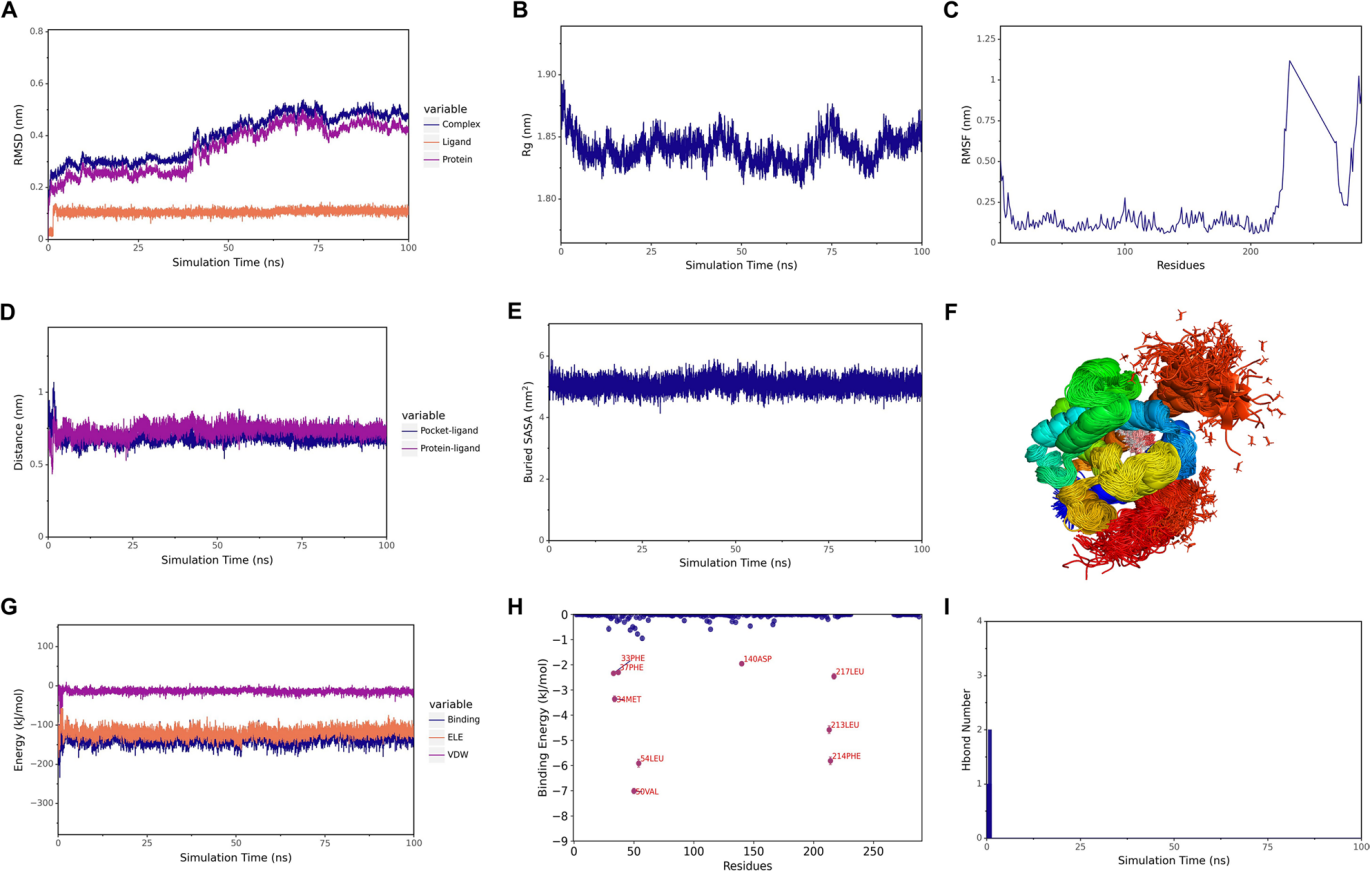
The molecular dynamics simulation. **A** RMSD analysis. **B** Rganalysis. **C** RMSF analysis. **D** The distance of the proteins and the small molecules binding site. **E** SASA analysis. **F** The superposition of simulated conformation. **G** The binding energy. **H** The amino acid binding energy contribution. **I** Hydrogen bond number

**Fig. 6:**
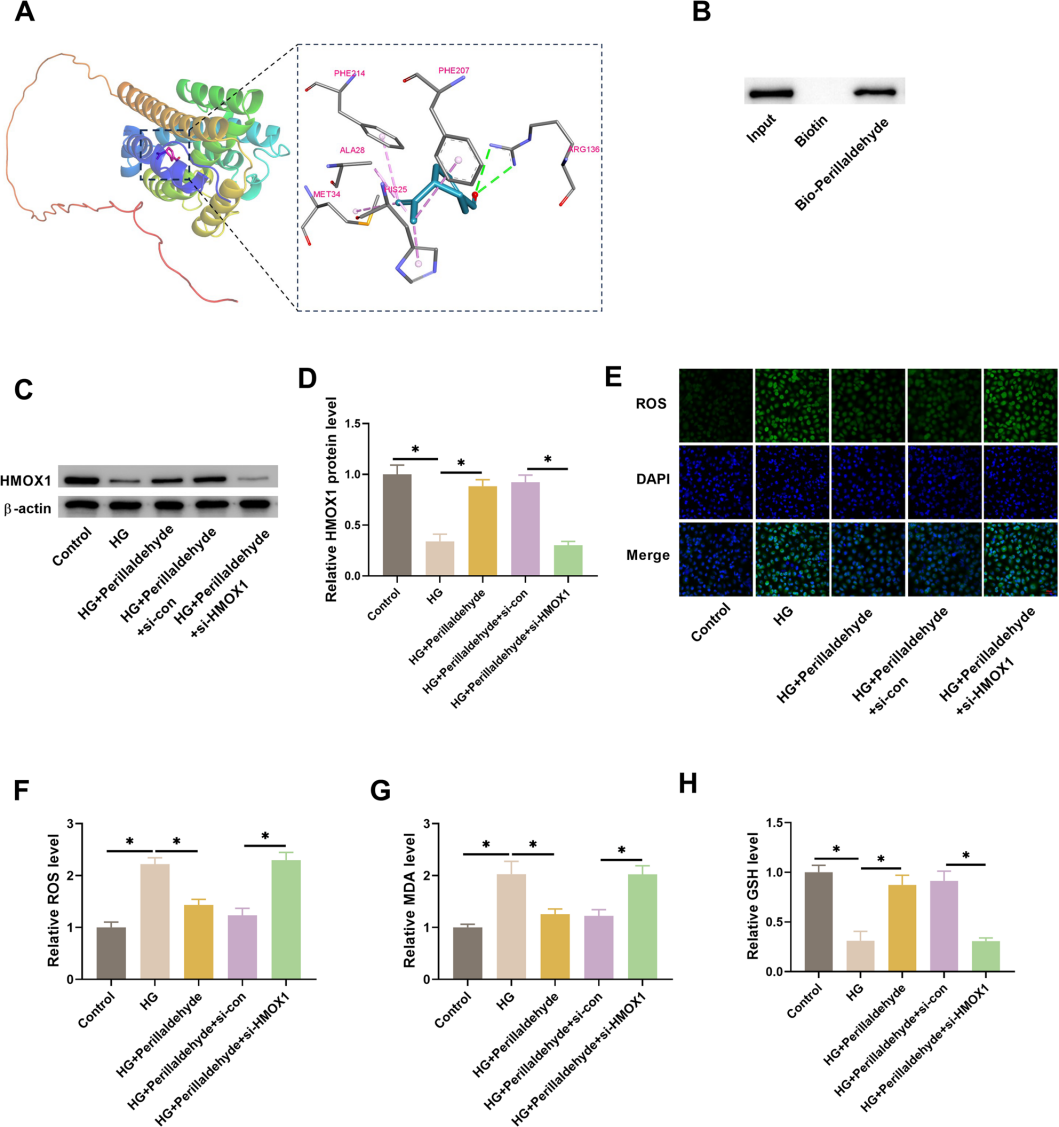
Perillaldehyde inhibits ROS-driven oxidative stress by increasing HMOX1. **A** Molecular docking of perillaldehyde with HMOX1 structure. HK-2 cells were treated with 80 µM of perillaldehyde. **B** The binding between perillaldehyde and HMOX1 by pull-down assay. **C**-**D** Perillaldehyde upregulated the expression of HMOX1 in HG-treated HK-2 cells. **E**-**H** The inhibitory effect of perillaldehyde on HG-treated oxidative stress was reversed by si-HMOX1, with increased ROS and MDA levels, and decreased GSH level. Scale bar = 20 μm. *N* = 3. *p < 0.05 vs. between indicated groups

The results of the molecular dynamics simulation suggested the good stability between perillaldehyde and HMOX1 (Fig. 5A-G). Furthermore, the binding contribution of amino acid residue was presented in Fig. 5H, with Val-50 and Leu-54 as key residues. The hydrogen bond numbers were mainly ranged from 0 to 2, indicating the low stability of hydrogen bonds (Fig. 5I). Molecular docking revealed the stable binding abilities of perillaldehyde and HMOX1 with a binding energy of -5.5 kcal/mol (Fig. 6A). The direct binding was validated by pull down assay (Fig. 6B). Based on the results that HMOX1 is involved in DN and closely related to both oxidative stress and inflammation (Fig. 6D), we speculated that perillaldehyde might exert its roles via regulating HMOX1. The 80 µM of perillaldehyde with highest efficacy was selected for further analyses. Our results showed that HMOX1 expression was significantly downregulated in HG-treated HK-2 cells, which could be elevated by perillaldehyde. However, transfection with si-HMOX1 prevented the perillaldehyde-caused increase in HMOX1 expression (Figs. 6C-D). In addition, the inhibitory effect of perillaldehyde on oxidative stress in HG-treated HK-2 cells was reversed by si-HMOX1, as shown by increased ROS generation and MDA content, and decreased GSH level (Figs. 6E-H). HMOX1 overexpression attenuated HG-induced ROS generation (Supplementary Fig. 1A-D).

### HMOX1 mediates the effect of perillaldehyde on NLRP3-mediated inflammation

**Fig. 7:**
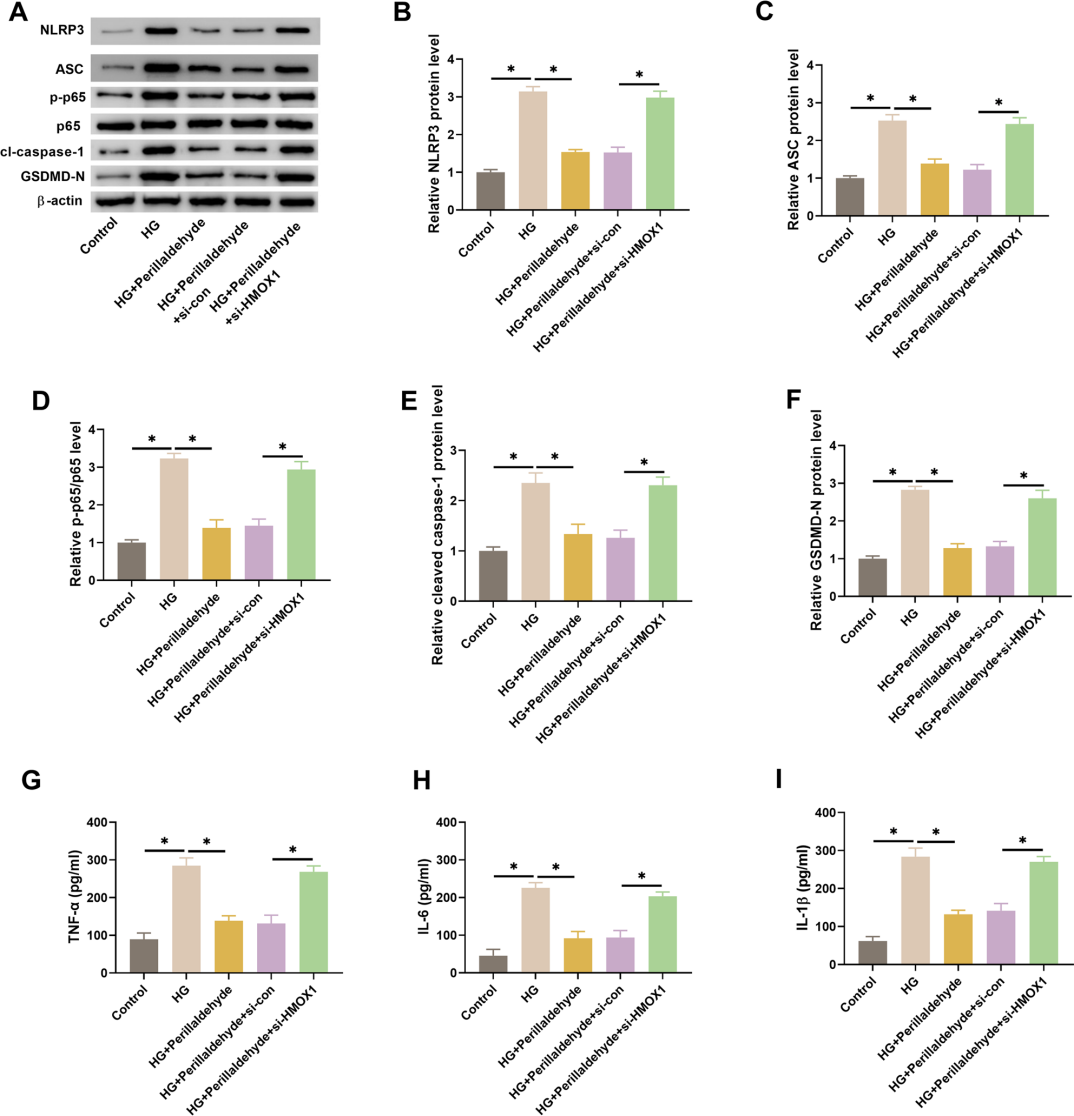
HMOX1 knockdown reverses the effect of perillaldehyde on NLRP3-mediated inflammation in HG-treated HK-2 cells. HK-2 cells were treated with 80 µM of perillaldehyde. **A**-**F** HMOX1 knockdown elevated the expression levels of NLRP3, ASC, cleaved caspase-1, GSDMD-N, and p-p65/p65 in perillaldehyde-treated HK-2 cells under HG condition. **G**-**I** HMOX1 knockdown induced the secretion levels of TNF-α, IL-6, and IL-1β in perillaldehyde-treated HK-2 cells under HG condition. *N* = 3. *p < 0.05 vs. between indicated groups

As shown in Figs. 7A-F, the inhibitory effects of perillaldehyde on HG-induced NLRP3, ASC, cleaved caspase-1, GSDMD-N and p-p65 expression were reversed by si-HMOX1. Knockdown of HMOX1 also elevated the levels of TNF-α, IL-6, and IL-1β in perillaldehyde-treated HK-2 cells under HG condition (Figs. 7G-I). HMOX1 overexpression attenuated HG-induced secretion of inflammatory factors (Supplementary Fig. 1E-F). These results indicated that perillaldehyde suppressed NLRP3-mediated inflammation via regulating HMOX1.

### HMOX1 mediates the effect of perillaldehyde on fibrosis in HG-treated HK-2 cells

**Fig. 8:**
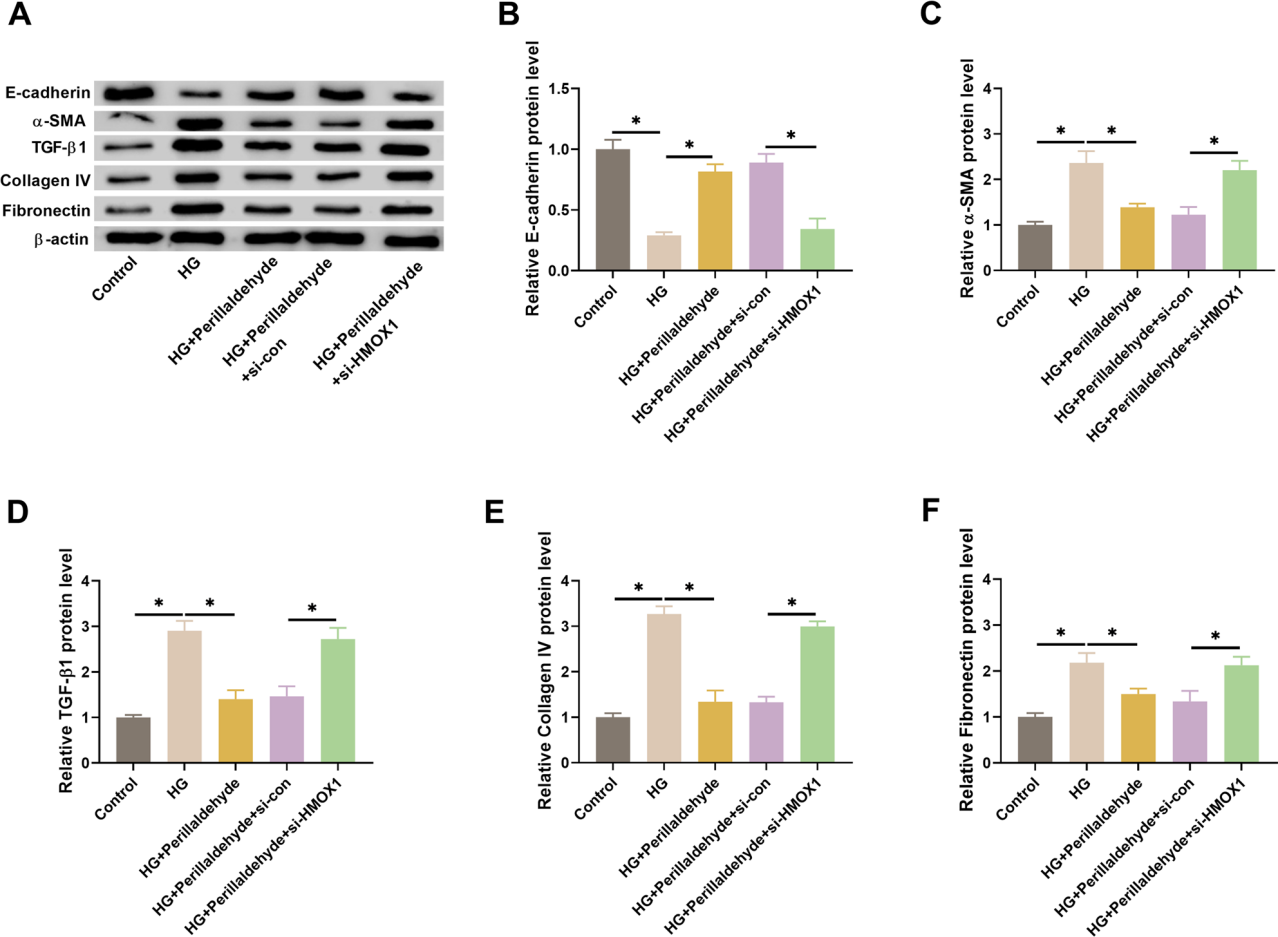
HMOX1 knockdown reverses the inhibitory effect of perillaldehyde on fibrosis in HG-treated HK-2 cells. HK-2 cells were treated with 80 µM of perillaldehyde. **A** Western blot images for the expression levels of fibrosis-related proteins including E-cadherin, α-SMA, TGF-β1, collagen IV, and fibronectin. **B**-**F** Relative expression levels of E-cadherin, α-SMA, TGF-β1, collagen IV, and fibronectin. *N* = 3. *p < 0.05 vs. between indicated groups

HMOX1 silencing attenuated the effects of perillaldehyde on expression levels of E-cadherin, α-SMA, TGF-β1, collagen IV, and fibronectin (Figs. 8A-F). These findings suggested that perillaldehyde reversed HG-induced fibrosis in HK-2 cells via regulating HMOX1.

### Perillaldehyde attenuates kidney injury in HFD/STZ-treated mice

**Fig. 9:**
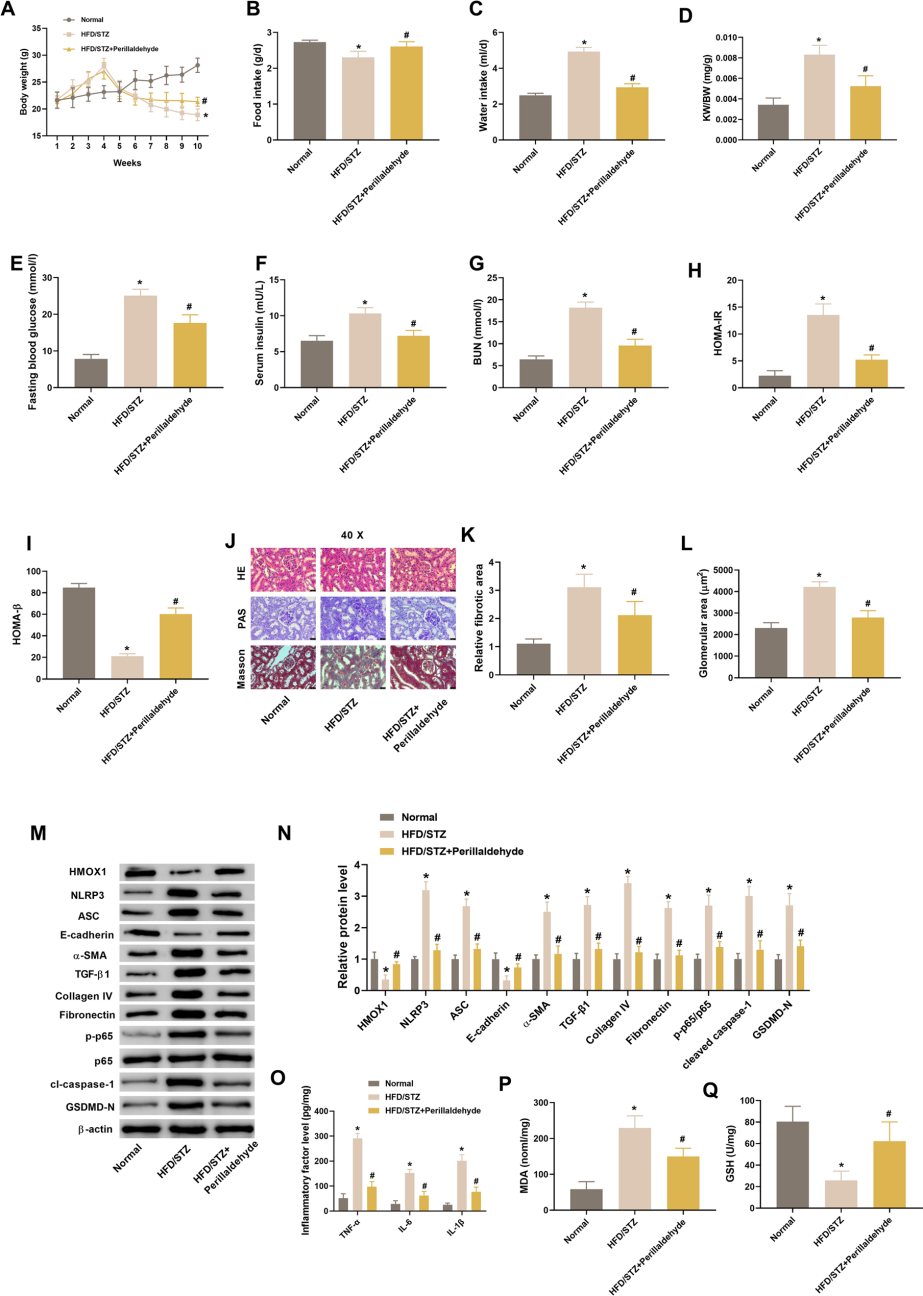
Perillaldehyde attenuates kidney injury in HFD/STZ-treated mice. **A** Body weight. **B**-**C** Food and water intake. **D** Kidney weight (KW)/body weight (BW) index. **E** Fasting blood glucose levels. **F** Serum insulin. **G** Blood urea nitrogen (BUN) levels. **H**-**I** HOMA-IR and HOMA-β. **J** Representative images of HE staining, Masson staining and PAS staining of renal tissues. **K**-**L** Relative renal fibrotic area and glomerular area. **M**-**N** Expression levels of HMOX1, NLRP3, ASC, E-cadherin, α-SMA, TGF-β1, collagen IV, fibronectin, cleaved caspase-1, GSDMD-N, and p-p65/p65 in kidney tissues. **O** Levels of TNF-α, IL-6, and IL-1β in kidney tissues. **P**-**Q** Levels of MDA and GSH in kidney tissues. *N* = 8. *p < 0.05 vs. normal control group, ^#^*p* < 0.05 vs. HFD/STZ group

Finally, we utilized an HFD/STZ murine model to evaluate the protective effect of perillaldehyde against kidney injury. As expected, HFD/STZ mice displayed significant increase in water intake, KW/BW ratio, fasting blood glucose, serum insulin, BUN, and HOMA-IR, and decrease in body weight, food intake, and HOMA-β, which could be prevented by perillaldehyde administration (Fig. 9A-I). Histopathological staining assays showed that HFD/STZ mice exhibited significant basement membrane thickening, expanded glomerular volume, renal fibrosis accumulation and mesangial matrix expansion (Fig. 9J, supplementary Fig. 2). Fibrotic and glomerular areas were markedly increased in HFD/STZ mice, while perillaldehyde administration lessened the fibrotic area (Fig. 9K-L). In consistent in vitro assays, western blot also proved that pretreatment with perillaldehyde caused significant increase in HMOX1 and E-cadherin expression, as well as decrease in NLRP3, ASC, cleaved caspase-1, GSDMD-N, p-p65, α-SMA, TGF-β1, collagen IV, and fibronectin expression (Figs. 9M-N). Perillaldehyde also exerted antioxidant and anti-inflammatory effects on kidneys from HFD/STZ mice, as shown by decreased MDA level, increased GSH level, as well as decreased levels of TNF-α, IL-6, and IL-1β (Figs. 9O-Q). These results suggested that pretreatment with perillaldehyde displayed renoprotection effect in HFD/STZ mice through its antioxidant, anti-inflammatory and anti-fibrotic activities.

## Discussion

Considerable recent evidence suggests that hyperglycemia can upregulate the ROS generation, which may be attributed to altered glucose metabolism, endothelial dysfunction, and damage of pancreatic β-cells [35]. The overproduction of ROS further causes cellular reduction-oxidation (redox) imbalance and ultimately leads to oxidative stress, and therefore aggravating the ROS production [36]. Modern therapeutic strategies have aimed at developing new approaches for proper treatment of hyperglycemia or inhibiting ROS overproduction to delay and prevent the onset of DM and its complications [37]. Previous studies reveal that perillaldehyde has inhibitory effect on ROS production in different pathological conditions. Perillaldehyde has been found to inhibit excessive accumulation of ROS and MDA content, as well as elevate the activity of antioxidant enzymes in vulvovaginal candidiasis (VVC) mice [19]. Perillaldehyde is capable of inhibiting ROS production and inducing the activation of antioxidant signaling in keratinocytes [15]. Here we found that treatment with perillaldehyde significantly decreased the ROS level and MDA content, and increased GSH level in HG-treated HK-2 cells, indicating that perillaldehyde exerted antioxidant activity in the in vitro DN model.

Except for the direct damage induced by oxidative stress, indirectly, it is believed to activate other pathogenic pathways. Hyperglycemic-induced oxidative stress can trigger the inflammatory response through increasing the levels of pro-inflammatory proteins such as NF-κB, NLRP3, IL-1β, IL-6, and TNF-α [38]. These factors then induce the differentiation and recruitment of immune-inflammatory cells, in which a great deal of inflammatory cytokines are secreted, leading to local and systemic inflammation [39]. NLRP3 inflammasome is a multimeric protein complex that remains one of the main aspects of inflammatory mechanism closely related to many diabetic diseases, such as DN [39]. It is apparent that perillaldehyde possesses a strong anti-inflammatory effect, as described in accumulating published studies. Perillaldehyde ameliorates intestinal inflammation in a dextran sulfate sodium (DSS)-induced colitis mouse model, as well as LPS-treated macrophage RAW264.7 cells [40]. Perillaldehyde can effectively alleviate inflammation in LPS-induced acute lung injury, as shown by improved lung histological changes, reduced production of inflammatory cytokines, and inflammatory cells infiltration [16]. A recent study has demonstrated that perillaldehyde attenuates NLRP3-mediated inflammation in an in vitro osteoarthritis (OA) model [41]. Here we found that perillaldehyde suppressed NLRP3-mediated inflammation and decreased the levels of TNF-α, IL-6, and IL-1β in HG-treated HK-2 cells.

Additionally, there is increasing evidence indicating that oxidative stress, inflammation and fibrosis are closely linked in the development and progression of DN, disrupting the renal structure and function [1]. It is becoming clear that inflammation has a crucial role in the initiation of renal fibrogenesis [42]. The infiltration of inflammatory cells, including dendritic cells, lymphocytes, mast cells, and monocytes/macrophages induce the production of growth factors and fibrogenic cytokines within the local microenvironment [42]. Next, the tubular epithelial cells and fibroblasts undergo phenotypic activation and/or transition to produce a large amount of extracellular matrix (ECM) components, contributing to the progression of fibrosis [42]. Together, the oxidative, inflammatory and fibrogenic events ultimately result in the destruction of renal parenchyma and loss of kidney function. A previous report suggested NLRP3 inflammasome activation was associated with inflammation and fibrosis in DN [27]. Perillaldehyde has been previously documented to alleviate liver tissue fibrosis and myocardial fibrosis [22, 43]. Our results indicated that perillaldehyde prevented the fibrogenic event in HG-treated HK-2 cells, as evidenced by changed expression levels of fibrosis-related proteins. In vivo assays also proved that pretreatment with perillaldehyde displayed renoprotection effect in HFD/STZ mice through its antioxidant, anti-inflammatory and anti-fibrotic activities. Taken together, we proved that perillaldehyde might be beneficial for DN through inhibiting oxidative stress, inflammation and fibrosis.

Previous studies prove that induction of HO-1 appears to reduce excessive ROS generation [44, 45]. Unsurprisingly, activation of HO-1 has become an attractive strategy for many oxidative stress-related diseases through preventing ROS-driven oxidative stress [25]. It has been demonstrated that perillaldehyde ameliorates Asperfillus fumigatus keratitis infection via inducing the activation of Nrf2/HO-1 signaling pathway [46]. Besides, the antioxidant activity of perillaldehyde in human keratinocytes is mediated by the Nrf2/HO-1 axis [15]. Perillaldehyde alleviates oxidative stress and inflammation by activating Nrf2/HO-1 signaling pathway in a spinal cord ischemia-reperfusion injury model [47]. However, there is no evidence for the direct interaction between perillaldehyde and HO-1. Here we found that perillaldehyde directly targeted HMOX1 and regulated its expression in HK-2 cells. Besides, we also observed that HMOX1 was involved in DN, and associated with both fibrosis and inflammation, which was also consistent to previous reports [26, 27]. Our study confirmed NAC could regulate fibrosis and inflammation, indicating HMOX1-mediated ROS might a key factor involved in the two processes. NLRP3 inflammasome was activated by high glucose through multiple signals, such as Smad3, and SIRT1 [48, 49]. This study confirmed high glucose could induced NLRP3 inflammasome activation through HMOX1-mediated ROS. Furthermore, knockdown of HMOX1 prevented the effects of perillaldehyde on oxidative stress, inflammation, and fibrosis in HG-treated HK-2 cells, implying that perillaldehyde exerted its positive effect on DN via regulating HMOX1. Targeting HMOX1 or potential effectors might provide novel strategies for anti-DN therapy.

However, there are some limitations in the current research. Firstly, this study investigated the roles of perillaldehyde in vitro and in vivo, but more works should be performed for potential human application in the future. Secondly, the grade-wise expression patterns of NLRP3 and HMOX1 should be explored for better adding the clinical relevance in the future. Moreover, the fibrosis is a multifactorial process, and the relationship among inflammation, oxidative stress, and fibrosis are not explored, which would be analyzed in future. The information on pharmacokinetic properties is still lacking, which might impact the clinical application.

In conclusion, our study clearly demonstrated that perillaldehyde exerted antioxidant, anti-inflammatory and anti-fibrotic activities in both HFD/STZ mice and HG-treated HK-2 cells. The positive effect of perillaldehyde could be mediated by HMOX1, which might be not the only one target. The dysfunction of HK-2 cells was associated with kidney damage. Considering the beneficial effect of perillaldehyde in DN, it might serve as a potential application in treating or preventing DN.

## Supplementary Information

Supplementary Material 1: Supplementary Figure 1. HMOX1 overexpression attenuates HG-induced injury. (A-B) HMOX1 expression was detected by western blot. (C-D) ROS level was measured in cells. Scale bar = 20 μm. (E-F) Levels of TNF-α, and IL-1β in cells. N=3. *p < 0.05 vs. between indicated groups

Supplementary Material 2: Supplementary Figure 2. Representative images of HE staining, Masson staining and PAS staining of renal tissues

## Data Availability

All data used during the current study are available from the corresponding author on reasonable request.
