# Supplementary figures and images for: Perillaldehyde inhibits oxidative stress, NLRP3-mediated inflammation and fibrosis in diabetic nephropathy through regulating HMOX1
Source: Hereditas. 2026 Mar 6;163:49. doi: 10.1186/s41065-026-00657-y (PMC13077860; doi:10.1186/s41065-026-00657-y)

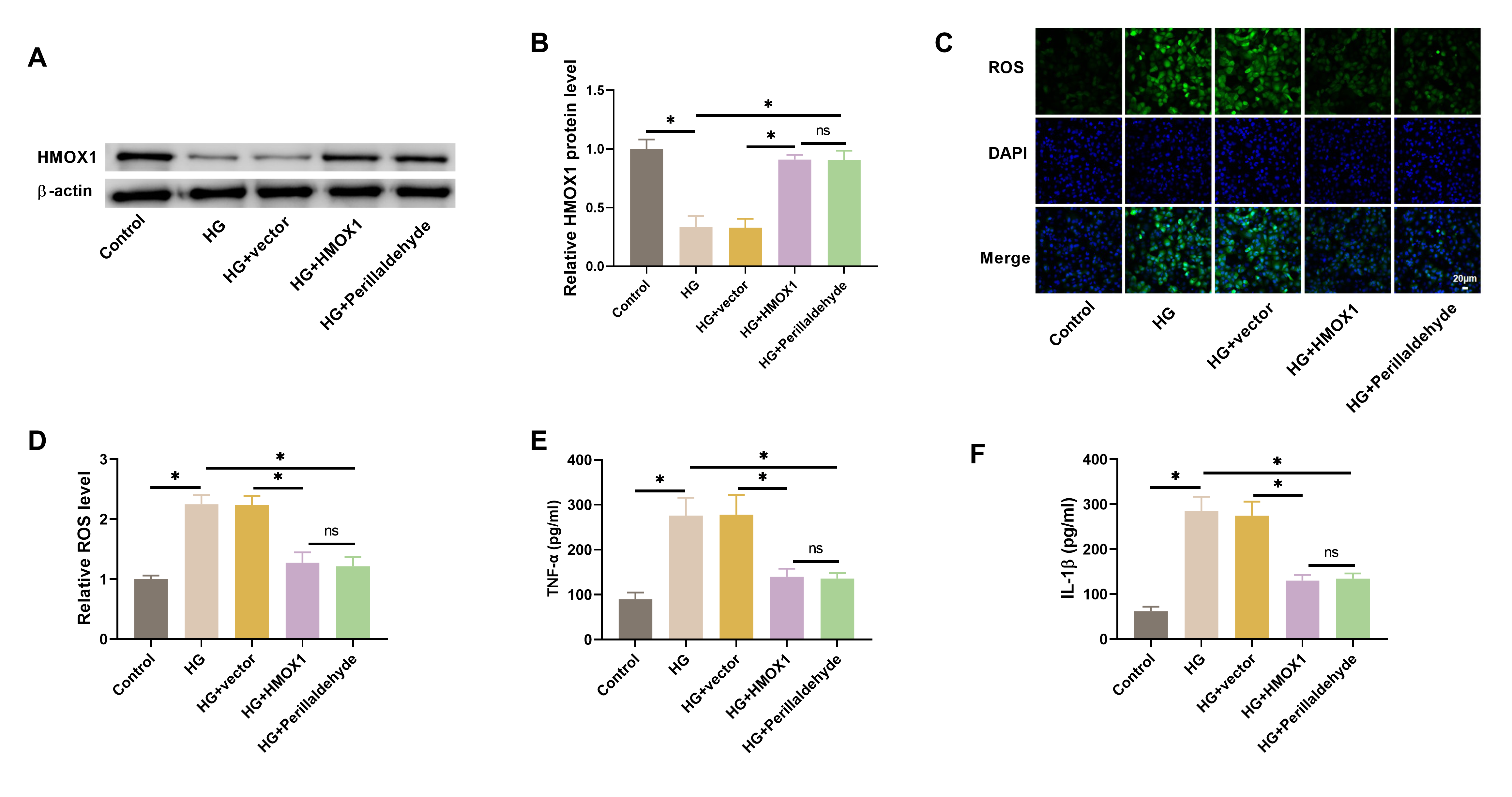

Supplement: Supplementary file 1 — Supplementary Material 1: Supplementary Figure 1. HMOX1 overexpression attenuates HG-induced injury. (A-B) HMOX1 expression was detected by western blot. (C-D) ROS level was measured in cells. Scale bar = 20 μm. (E-F) Levels of TNF-α, and IL-1β in cells. N=3. *p < 0.05 vs. between indicated groups [file 41065_2026_657_MOESM1_ESM.tif]

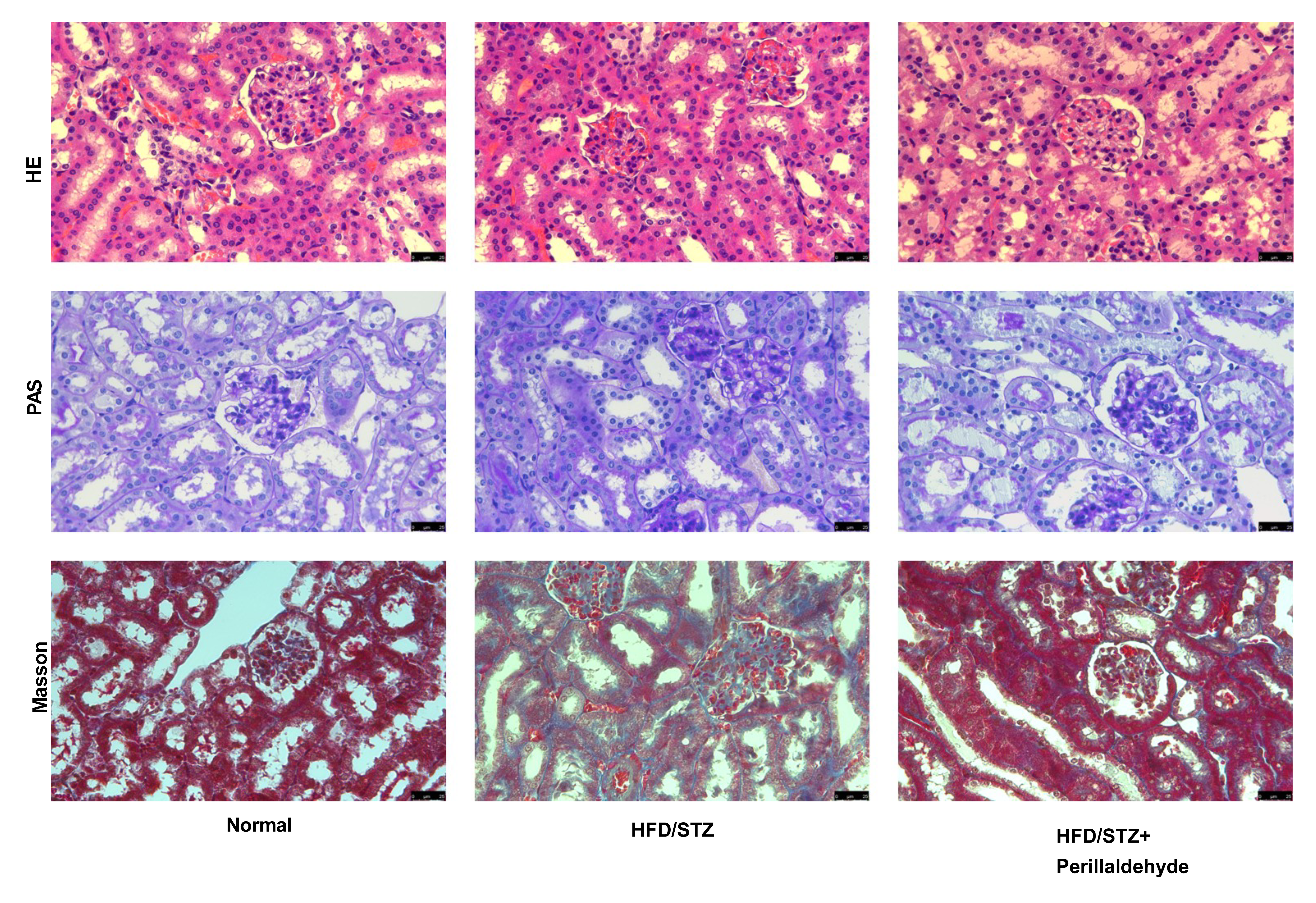

Supplement: Supplementary file 2 — Supplementary Material 2: Supplementary Figure 2. Representative images of HE staining, Masson staining and PAS staining of renal tissues [file 41065_2026_657_MOESM2_ESM.tif]
